# Genome-wide Methylomic Analysis of Monozygotic Twins Discordant for Adolescent Depression

**DOI:** 10.1016/j.biopsych.2014.04.013

**Published:** 2014-12-15

**Authors:** Emma L. Dempster, Chloe C.Y. Wong, Kathryn J. Lester, Joe Burrage, Alice M. Gregory, Jonathan Mill, Thalia C. Eley

**Affiliations:** a University of Exeter Medical School, Exeter University, Exeter; b Social Genetic Developmental Psychiatry Centre, Institute of Psychiatry, King’s College London; c Department of Psychology, Goldsmiths, University of London, London, United Kingdom.

**Keywords:** Adolescent depression, depression, DNA methylation, epigenetic, genomics, monozygotic twins

## Abstract

**Background:**

Adolescent depression is a common neuropsychiatric disorder that often continues into adulthood and is associated with a wide range of poor outcomes including suicide. Although numerous studies have looked at genetic markers associated with depression, the role of epigenetic variation remains relatively unexplored.

**Methods:**

Monozygotic (MZ) twins were selected from an adolescent twin study designed to investigate the interplay of genetic and environmental factors in the development of emotional and behavioral difficulties. There were 18 pairs of MZ twins identified in which one member scored consistently higher (group mean within the clinically significant range) on self-rated depression than the other. We assessed genome-wide patterns of DNA methylation in twin buccal cell DNA using the Infinium HumanMethylation450 BeadChip from Illumina. Quality control and data preprocessing was undertaken using the wateRmelon package. Differentially methylated probes (DMPs) were identified using an analysis strategy taking into account both the significance and the magnitude of DNA methylation differences. The top differentially methylated DMP was successfully validated by bisulfite-pyrosequencing, and identified DMPs were tested in postmortem brain samples obtained from patients with major depressive disorder (*n* = 14) and matched control subjects (*n* = 15).

**Results:**

Two reproducible depression-associated DMPs were identified, including the top-ranked DMP that was located within *STK32C*, which encodes a serine/threonine kinase, of unknown function.

**Conclusions:**

Our data indicate that DNA methylation differences are apparent in MZ twins discordant for adolescent depression and that some of the disease-associated variation observed in buccal cell DNA is mirrored in adult brain tissue obtained from individuals with clinical depression.

Depression is a common chronic psychiatric disorder characterized by episodes of severe low mood. Depression ranks second in global disease burden (1), but despite extensive research, its etiology is not well understood. The heritability of depression is estimated to be 31%–42% (2), although genome-wide association studies have been unable to identify reproducible association signals (3). Although some of this lack of replication in genetic studies can be explained by the heterogeneous nature of depression, increased understanding about the functional complexity of the genome has led to growing recognition about the likely role of non–sequence-based variation in disease etiology (4, 5).

Despite the apparently high heritability of depression, there is considerable discordance within monozygotic (MZ) twin pairs (6), implicating a role for nongenetic, presumably environmental, factors. One mechanism by which the environment can have an impact on individual development is via epigenetic changes to gene expression. Epigenetic mechanisms mediate developmental changes in gene function independent of DNA sequence variation, principally through alterations in DNA methylation and chromatin structure (7). Epigenetic processes have been shown to underlie much of the plasticity in the mature central nervous system, playing a key role in learning and memory and enabling the brain to respond to environmental stimuli (8). Accumulating evidence from animal models indicates that many environmental stressors in early life associated with an increased risk of depression can precipitate epigenetic changes in the brain (9), although most research has focused on a single gene or functional pathway (10, 11, 12, 13). Emerging evidence implicates a role for epigenetic modifications across the genome in several human neuropsychiatric disorders, including depression (4). Although few empirical studies have systematically examined the role of altered epigenetic processes in depression, more recent analyses provide evidence for altered DNA methylation and histone modifications in the disorder (14, 15, 16, 17).

Adolescence is a unique period characterized by both physical and emotional development and exposure to novel environmental stressors (18). It is a period of active brain maturation, characterized by processes such as synaptic pruning (19), and is the developmental stage when the signs of many psychiatric disorders first manifest (20). The prevalence of depression dramatically increases during adolescence, particularly in girls (21). Once established, adolescent depression commonly continues into adulthood and is associated with a wide range of poor outcomes including suicide (22, 23).

To identify epigenetic variation associated with adolescent depression, we examined genome-wide patterns of DNA methylation in buccal cell–derived DNA from MZ twin pairs discordant for adolescent depression symptoms assessed at multiple time points. The use of disease-discordant MZ twins is a powerful strategy in epigenetic epidemiology because identical twins are matched for genotype, age, sex, maternal environment, population cohort effects, and exposure to many shared environmental factors (24, 25). DNA methylation differences have been associated with MZ twin discordance for several complex phenotypic traits, including psychosis (26), autism (27), and type 1 diabetes (28). In the context of depression, the use of adolescent twins has the advantage that DNA methylation is less likely to be influenced by other environmental factors, lifestyle choices, and medication exposure, which can confound studies in older clinical cohorts.

## Methods and Materials

### Sample Description

This study was nested within the Genesis 12–19 (G1219) Study, an adolescent twin and sibling study (*N* = 1300 pairs) designed to investigate the development of emotional and behavioral difficulties focusing on the interplay of genes and environment (18). The twins were recruited via the United Kingdom Office of National Statistics, and four waves of data have been collected to date. At each wave, participants completed the Short Mood and Feelings Questionnaire (SMFQ) (29), a self-report measure of depression symptoms. Scores on this measure range from 0–26 (mean, 7). The mean difference score within the Genesis 12–19 Study twin/sibling pairs at each wave ranged from 4.06–5.13 (SD, 3.99–4.80). We designated a difference of 6 (i.e., approximately 1.5 times the SD of the difference) as representing sufficient discordancy to be of interest for the current study and identified 18 MZ twin pairs (13 female pairs, 5 male pairs, all Caucasian) with consistent discordancy on at least two measurement occasions. The mean SMFQ score for each of the twin pair members with depression was 12.38 [>1 SD above the clinical threshold of 8 (29)], and the cotwin group mean was 6.42. Twins did not differ significantly on number or severity of self-reported life events at any of the assessment time points. Because this was a population-based sample, DNA was obtained from buccal cells (mean age, 16.8 years; SD, 2.4), and DNA was isolated using a standard protocol (30).

### Methylomic Profiling

DNA was converted with sodium bisulfite in duplicate using the EZ-96 DNA methylation kit (Zymo Research Corporation, Irvine, California) and profiled using the Infinium HumanMethylation450 BeadChip (Illumina, Inc, San Diego, California) as previously described (30). Each twin pair was processed together on the same array to negate batch effects. Raw data were preprocessed using GenomeStudio software (Illumina, Inc). Stringent quality control checks, quantile normalization, and separate background adjustment of methylated and unmethylated intensities of type I and II probes were implemented using the wateRmelon package in R (available from the bioConductor repository www.bioconductor.org) (30). Only samples with <5% of sites with a detection *p* value < .05 were included, and probes with >5% of samples with a detection *p* value < .05 or a bead count <3 in 5% of samples were removed from the analysis. The final analysis included 439,846 probes, and all samples passed our stringent quality control filter. Polymorphic single nucleotide polymorphism control probes located on the array were used to confirm that all twin pairs were monozygous. With the aim of identifying real, biologically relevant within-twin and between-group DNA methylation differences, we used an analytic approach that incorporates both the significance (i.e., paired *t* test statistic) and the magnitude (i.e., absolute Δβ) of any observed differences to produce a ranked list of differentially methylated probes (DMPs) (26, 27). Briefly, probes were ranked separately by paired *t* test *p* value and Δβ, and the ranks were summed (Figure S1 in Supplement 1). An *F* test was used to examine whether the variances between affected and unaffected groups for each individual probe differed. To test the overall mean variance across all probes between the two groups, the variance was calculated for each probe in each group separately, and the resulting distributions were compared using the Wilcoxon signed rank test. Region level analysis was performed using the Bioconductor package Illumina Methylation Analyzer (31). Genes were assigned to probes using the Genomic Regions Enrichment of Annotations Tool (GREAT) package from the Bejerano Lab at Stanford University (http://bejerano.stanford.edu/great/public/html) (32) taking into account the functional significance of *cis*-regulatory regions. Pathway and network analysis was performed using Ingenuity Pathway Analysis (Ingenuity Systems, Redwood City, California). Enrichment for specific pathways and biological functions was determined relative to the proprietary database, using a right-tailed Fisher’s Exact Test at a significance level of *p* < .05.

### DMP Validation Using Bisulfite Pyrosequencing

We performed independent verification analyses on the highest ranked depression-associated probe (cg07080019, *STK32C*) nominated from the genome-wide microarray analysis using bisulfite pyrosequencing. The assay spanned multiple CpG sites, including the specific CpG interrogated on the Infinium HumanMethylation450 BeadChip array. Briefly, 500 ng DNA from each individual was independently treated with sodium bisulfite in duplicate using the EZ 96-DNA methylation kit as described earlier. Bisulfite–polymerase chain reaction amplification was performed in duplicate, and quantitative DNA methylation analysis was conducted using the PyroMark Q24 pyrosequencer (Qiagen, Valencia, California) (Table S1 in Supplement 1).

### Replication in Postmortem Brain Tissue

Postmortem cerebellum samples were obtained from the Stanley Brain Collection at the Stanley Medical Research Institute (Rockville, Maryland) (33) from individuals with a diagnosis of major depressive disorder (MDD) (*n* = 14) and from unaffected individuals with no diagnosed psychiatric disorder (*n* = 15) as control samples. Detailed demographic information for these samples is given in Table S2 in Supplement 1. DNA was isolated from ~70 mg of tissue using a standard phenol-chloroform extraction protocol, sodium bisulfite treated in duplicate, and profiled using the Infinium HumanMethylation450 BeadChip as described earlier. Cases and controls were randomly assigned and run as a single batch to minimize any technical confounding. DNA methylation data for specific probes nominated from our discordant MZ twin analysis were assessed for an association with major depression.

## Results

### Site-Specific DNA Differences Are Widespread in MZ Twins Discordant for Depression

**Figure 1:**
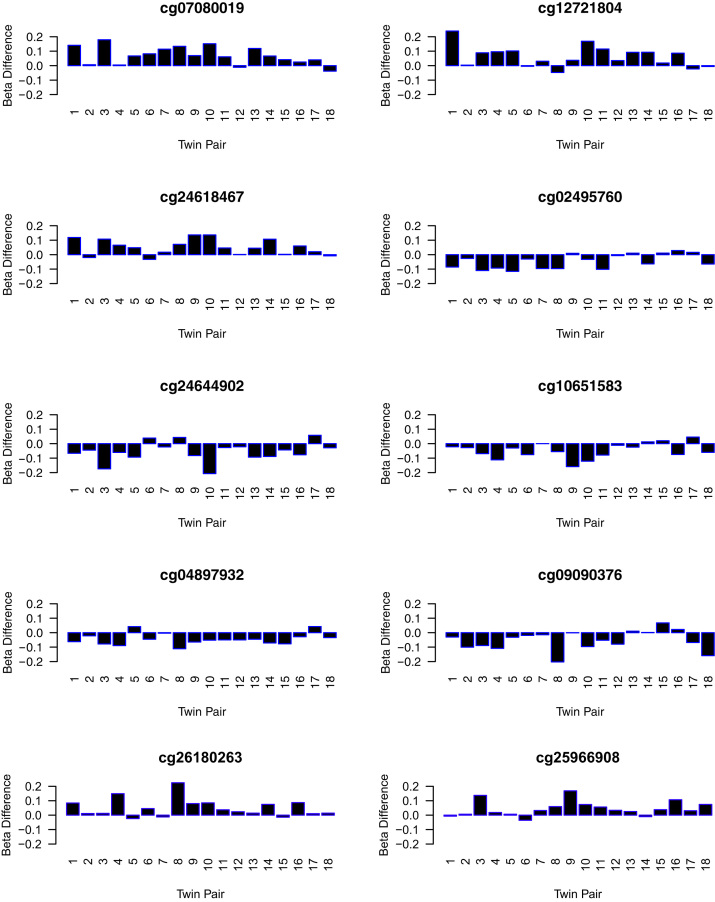
Difference in DNA methylation (β value) between twins for the eight top-ranked probes (twin with depression–cotwin without depression).

**Figure 2:**
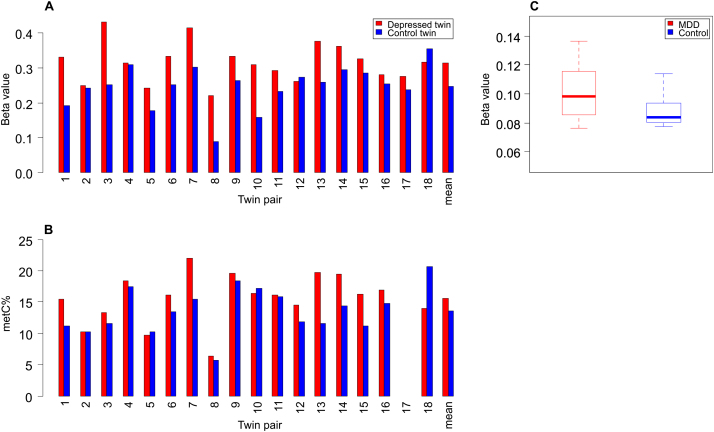
Validation and cross-tissue replication of the top-ranked differentially methylated probe (cg07080019) via bisulfite pyrosequencing. Infinium HumanMethylation450 BeadChip array **(A)** and bisulfite-pyrosequencing **(B)** data for cg07080019 across each discordant twin pair. Methylation levels estimated by pyrosequencing are slightly lower than the array but confirm depression-associated hypermethylation (*p* = .02). One twin pair (pair 17) was removed from analyses because of failed amplification. **(C)** Group comparison of DNA methylation at cg07080019 in the postmortem cerebellum samples confirms depression-associated hypermethylation (*p* = .03). MDD, major depressive disorder; metC%, percentage cytosine methylation.

Using an analysis method designed to identify the largest and most significant differences in DNA methylation at individual CpG sites, we identified multiple CpG sites across the genome exhibiting significant depression-associated differential DNA methylation. The 10 top-ranked DMPs are shown in Table 1 and Figure 1 (the 100 top-ranked DMPs are listed in Table S3 in Supplement 1). The top ranked DMP (cg07080019, Δβ = +.07, *p* = .0001) is located in the eighth intron of *STK32C* on chromosome 10q36.3, encoding a serine/threonine kinase of unknown function that is highly expressed in the brain. Depression-associated hypermethylation at this DMP was successfully validated by bisulfite pyrosequencing (*p* = .02) (Figure 2). DNA methylation at the three additional CpG sites assessed in our bisulfite-pyrosequencing assay was strongly correlated with cg07080019 (*r*^*2*^ > .98) with mean DNA methylation across the amplicon being significantly associated with depression (*p* = .01).

**Table 1:** Ten Top-Ranked DMPs in MZ Twins Discordant for Adolescent Depression

Rank	Probe	Mean Δβ	*p* Value	Genomic Coordinate (hg19)	Gene (Distance from TSS)
1	cg07080019	+.07	.000144	Chr10:134036804	*DPYSL4* (+36391), *STK32C* (+84672)
2	cg12721804	+.06	.001758	Chr8:29194923	*KIF13B* (−74314), *DUSP4* (+13343)
3	cg24618467	+.05	.000822	Chr2:11245514	*PQLC3* (−50025), *KCNF1* (+193452)
4	cg02495760	−.05	.000950	Chr12:48356074	*TMEM106C* (−1255)
5	cg24644902	−.06	.003038	Chr10:77036275	*COMTD1* (−40506), *ZNF503* (+125237)
6	cg10651583	−.05	.001586	Chr12:57941069	*KIF5A* (−2777), *DCTN2* (−92)
7	cg04897932	−.04	.000225	Chr17:8460923	*MYH10* (+73112), *NDEL1* (+121745)
8	cg09090376	−.05	.003880	Chr11:33040743	*DEPDC7* (+3334), *CSTF3* (+142293)
9	cg26180263	+.05	.003454	Chr14:58429389	*SLC35F4* (−96798), *C14orf37* (+189457)
10	cg25966908	+.05	.001861	Chr7:55516751	*LANCL2* (+83611), *VOPP1* (+123448)

Ranked by a combination of both mean absolute difference in methylation level and statistical significance.

DMPs, differentially methylated probes; MZ, monozygotic; TSS, transcription start site.

Many top-ranked DMPs are associated with genes that have been previously implicated in depression and other related neuropsychiatric disorders, including *DUSP4* (34), *NDEL1* (35), *ACP1* (36), *SSTR5* (37), *NR3C1* (38), and *GABRA3* (39). Network analysis of the 175 genes associated with the 100 top-ranked probes using Ingenuity Pathway Analysis highlighted significant enrichment of pathways involved in nervous system development and function, neurologic disease, and psychological disorders (Tables S4–S6 in Supplement 1). Nervous system development and function was the top-ranked biological function (*p* = 6.15E-05), including numerous genes ranked highly in our probe-wise analysis (e.g., *DPYSL4, NDEL1, MYH10, KIF5A*). Region level analysis using Illumina Methylation Analyzer identified several depression-associated differentially methylated regions (Table S2 in Supplement 1). The top-ranked differentially methylated region was a 300-bp region in the first intron of *PLCXD3,* encompassing four probes hypermethylated in affected twins (Δβ = −.024; *p* = .0005) (Figure S2 and Table S7 in Supplement 1). Four genes highlighted from the Illumina Methylation Analyzer regional analysis also were featured in the 100 top-ranked individual probes from the main analysis: *STK32C* (the overall top ranked probe), *DOK6* (ranked 16), *UBE2D3* (ranked 61), and *POLR2I* (ranked 72). None of the top-ranked probes showed significant differences between male and female samples.

### Several Top-Ranked DMPs Are Also Associated with MDD in the Cerebellum

**Figure 3:**
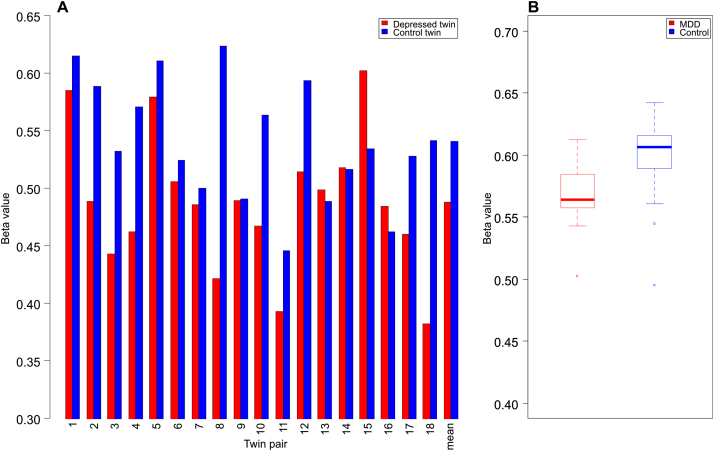
Replication of differentially methylated probe cg09090376 in cerebellum samples. **(A)** Probe cg09090376 shows consistent depression-associated hypomethylation across discordant monozygotic twin pairs (*p* = .004), which is **(B)** replicated in postmortem cerebellum samples (*p* = .03). MDD, major depressive disorder.

Two of the 10 top-ranked DMPs identified in buccal cell DNA from our discordant twins were also found to be significantly associated with MDD in postmortem cerebellum samples, including our top-ranked probe, cg07080019, which was found to be significantly hypermethylated in MDD cerebellum samples compared with matched control samples (average β difference = .02, *p* = .03) (Figure 2C). Probe cg09090376, mapping to the first intron of *DEPDC7* and ranked eighth in our discordant twin analysis, also exhibited significant differences in the postmortem brain samples consistent with differences observed in buccal cells from the discordant MZ twin pairs (average β difference = .03, *p* = .03) (Figure 3).

### There Is Increased Variability of DNA Methylation in Twins with Depression

As expected, no differences in overall mean genome-wide DNA methylation was seen between twins with depression and unaffected twins (*p* = .84). However, the difference in variance was significant *(p* < 4.58 × 10^−10^). Of the individual probes where the variance was significantly different (*p* < .05), 53% of probes showed a higher variance in the group with depression compared with the cotwin control group, which is consistent with a previous study on MZ twins discordant for MDD (14).

## Discussion

The present study is the first comprehensive analysis of DNA methylation differences in adolescent MZ twins discordant for self-reported depression using a genome-wide approach. The top-ranked DMP (cg07080019) is located in *STK32C*, with depression-associated hypermethylation at this locus being successfully validated using bisulfite pyrosequencing. We also show that the same CpG site is significantly hypermethylated in postmortem cerebellum samples from subjects with MDD compared with control subjects without a psychiatric diagnosis. The function of *STK32C* is unknown, but it is highly expressed in the brain (40). The presence of a low-frequency (minor allele frequency = 3.57%) C>T single nucleotide polymorphism (rs145220785) at cg07080019 is notable; although this variant could potentially confound case-control studies, it is not an issue for the MZ twin study design where both twins have the same genotype. Because the differentially methylated region in *STK32C* is intronic, it is uncertain whether a DNA methylation change in this region could alter gene expression or gene splicing. However, the interrogation of publicly available Encyclopedia of DNA Elements data (41) shows that this region is situated in a DNAase I hypersensitivity cluster and contains several transcription factor–binding sites suggesting that it resides in a transcriptionally active region of the genome, which could be functionally influenced by alterations in DNA methylation.

Several of the other top-ranked DMPs are located in the vicinity of genes that have previously been implicated in psychiatric disorders. For example, cg12721804, which is hypermethylated in affected twins (Δβ = +.06, *p* = .001), is located in the last exon of *DUSP4* (also known as mitogen-activated protein kinase phosphatase 2), which has been shown to be unregulated in the brains of individuals with depression who committed suicide (34). Similarly, cg04897932, which is hypomethylated in affected twins (Δβ = −.04, *p* = .0002), is located upstream of *NDEL1,* which forms a complex with *DISC1,* an established risk gene for several psychiatric conditions including major depression (35). Finally, cg24565620, which was hypomethylated in affected twins (Δβ = −.05, *p* = .004) is located upstream of *ACP1* (acid phosphatase 1), a tyrosine phosphatase that influences Wnt signaling, that has been implicated in suicide in genome-wide association studies and expression analyses (36). Several other genes of note were present in the 100 top-ranked probes, including *SSTR5* (37), *NR3C1* (38), and *GABRA3* (39). *NR3C1* is of particular interest given that epigenetic variation in this region has been associated with early-life stress in both humans and rodents (42).

A global approach looking across all probes on the array revealed no significant difference between twins with depression and their unaffected cotwins. However, the variance across β values in the twins with depression is significantly larger than in the cotwins, consistent with the findings of a study that looked at DNA methylation in 12 adult MZ twin pairs discordant for MDD (14). These data suggest that DNA methylation is more variable in individuals with depression, potentially indicating that the methylome of the group with depression is generally more reactive to external stimuli or stochastic processes. In our study, we found no significant differences in the number of stressful life events reported by twins with depression and unaffected cotwins.

There is little overlap in the genes associated with our top-ranked DMPs and the genes identified in other epigenetic studies of depression (14, 16, 17), although direct comparisons are difficult because of differences in study design, technology platforms, phenotypic analysis, tissue, and sample size. However, it is notable that Ingenuity Pathway Analysis network analysis highlighted an enrichment of genes involved in nervous system development and function among our depression-associated DMPs, which is consistent with previous reports (16, 17).

Despite the power of the discordant MZ twin approach for epigenetic epidemiology, there are several limitations to this study. First, because of the relatively small sample size and effect sizes detected, no probe reached Bonferroni-corrected levels of significance. However, DNA methylation studies in other psychiatric phenotypes (and complex disorders in general) report similarly small absolute differences (26, 27, 28), and, given the known nonindependence of DNA methylation across the probes represented on the array (43), it is likely that conventional methods of global statistical significance are not relevant to these analyses. Instead, we took steps to validate and replicate our results. Our analytic approach was to rank DMPs by both statistical significance and absolute difference, and we were able to validate our top-ranked DMP using bisulfite pyrosequencing and to replicate the association in brain tissue. A second caveat is that the genome-wide data generated in this study are from peripheral buccal cell DNA and not the target organ of depression, the brain. However, in the absence of availability of brain tissue from MZ twins discordant for depression, buccal swabs were selected as the most suitable peripheral tissue because they derive from the same embryonic cells as brain tissue (ectoderm) and have less cellular heterogeneity compared with blood (44). Although there are well-documented tissue-specific differences in DNA methylation (45), a study suggests that some disease epimutations may be detectable across multiple tissues (46). A related concern is that we had access only to cerebellum tissue, which may not be the optimal brain region to study in the context of depression. A third limitation of this study is that clinical diagnoses of depression were not used. Instead, we selected MZ pairs in which one twin scored >1.5 SDs higher on self-reported depressive ratings (SMFQ) than his or her cotwin on at least two occasions. A cutoff of 8 on this measure has been shown to have reliable predictive ability, with a sensitivity of 60% and specificity of 85% compared with depression diagnoses made by structured interview (29). The mean SMFQ score for our affected twins was 12.38, which strongly suggests that these individuals were experiencing a clinically significant level of depressive symptoms. However, it would be important to replicate these findings using a sample of clinically depressed adolescents. Although our sample contained more female than male twin pairs, we were not powered to look at sex effects. However, none of the top-ranked probes showed different levels of DNA methylation between male and female subjects. One final caveat in this study is that chorionicity data are not available on these twins, a potential limitation given that whether or not MZ twins share a placenta may influence transcriptional and epigenomic differences (47).

In conclusion, we have identified DNA methylation differences associated with adolescent depressive symptoms in peripheral DNA samples from discordant MZ twin pairs. Two of the top-ranked DMPs were found to be differentially methylated in postmortem brain tissue samples from individuals with MDD. Such trait-specific differentially methylated regions that are stable across tissues are of particular interest because they have potential use as biomarkers. Together, these data along with findings emerging from other studies of depression provide evidence for a role of epigenetic variation in the etiology of depression.
