# Supplementary material for: Genome-wide Methylomic Analysis of Monozygotic Twins Discordant for Adolescent Depression
Source: Biol Psychiatry. 2014 Dec 15;76(12):977–83. doi: 10.1016/j.biopsych.2014.04.013 (PMC4252163; doi:10.1016/j.biopsych.2014.04.013)
Supplement: Supplementary file 1 — Supplementary Material [file mmc1.pdf]

Genome-Wide Methylomic Analysis of Monozygotic Twins Discordant for Adolescent Depression

Supplemental Information

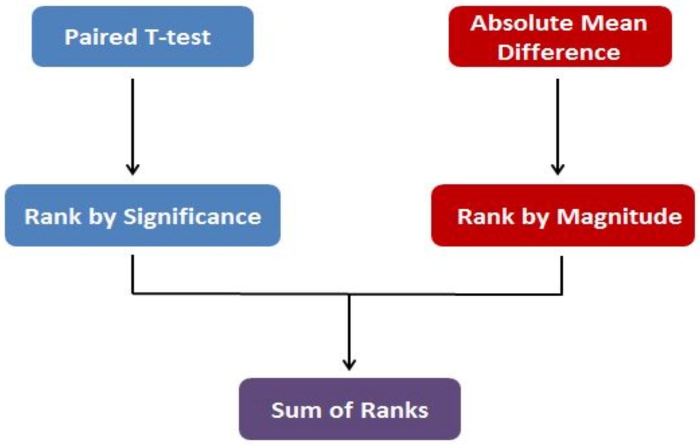

Figure S1. Overview of the analysis strategy used in this study. Probes were ranked separately by *p*-value and delta-beta, and the ranks summed. Probes were then ordered by the summed rank.

Table S1. Pyrosequencing assay details: Primers and assay conditions for the verification of the top probe cg07080019

Primer	Sequence	PCR Conditions
Forward Primer	ATTTGTAGGGAAGGTTTTGTTG	
Reverse Primer	[btn]ACTCATCTAACCTATAAAAATCCCATAAT	98°C 15 min
Sequencing Primer	ATTTGTTAGGAGGTGG	98°C 30 s } x 40 cycles
Target Sequence	TAGTYGAGGTTYGGTTAGYGGTTAATATTYGGTATATGGTTGGTTTTATAGTAATTATTTT	56°C 30 s } x 40 cycles
Genomic Sequence (Hg19)	(chr10:134,036,794-134,036,828)<br>AGCCGAGGCCCGGCTAGCGGCTAACACTCGGCACA	72°C 60 s } x 40 cycles

PCR, polymerase chain reaction.

Table S2. Demographic information and matched variables for the Stanley Foundation Neuropathology Consortium brain samples

	**Major Depression**	**Normal Controls**
Age (years), mean (range)	46.9 (30-65)	48.1 (29–68)
Sex	8M, 6F	9M, 6F
Race	14C	14C, 1AA
PMI (h), mean (range)	28.9 (23-47)	23.7 (8–42)
Brain pH, mean (range)	6.2 (5.8–6.4)	6.3 (5.8–6.6)
Suicide	6	0
Duration of Illness	12.75 years	N/A

AA, African American; C, Caucasian; F, female; M, male; PMI, postmortem interval.

Table S3. Top 100 ranked DMPs, ranked on *p*-value and magnitude of DNA methylation change

Rank	Probe ID	Depressed Twin Mean	Co-twin Mean	βΔ	*P* Value	Hg19	Illumina Gene Annotation	Probe Type	Gene Annotation from GREAT (Distance from TSS)	Flagged Probes^
1	cg07080019	0.32	0.25	0.07	1.44E-04	Chr10:134036804	STK32C	II	DPYSL4 (+36391), STK32C (+84672)	*
2	cg12721804	0.76	0.70	0.06	1.76E-03	Chr8:29194923	DUSP4	II	KIF13B (-74314), DUSP4 (+13343)	
3	cg24618467	0.80	0.75	0.05	8.22E-04	Chr2:11245514		I	PQLC3 (-50025), KCNF1 (+193452)	
4	cg02495760	0.63	0.68	-0.05	9.50E-04	Chr12:48356074	TMEM106C	II	TMEM106C (-1255)	
5	cg24644902	0.29	0.34	-0.06	3.04E-03	Chr10:77036275		I	COMTD1 (-40506), ZNF503 (+125237)	
6	cg10651583	0.26	0.31	-0.05	1.59E-03	Chr12:57941069	DCTN2	I	KIF5A (-2777), DCTN2 (-92)	
7	cg04897932	0.71	0.75	-0.04	2.25E-04	Chr17:8460923	MYH10	II	MYH10 (+73112), NDEL1 (+121745)	
8	cg09090376	0.49	0.54	-0.05	3.88E-03	Chr11:33040743	DEPDC7	II	DEPDC7 (+3334), CSTF3 (+142293)	
9	cg26180263	0.45	0.40	0.05	3.45E-03	Chr14:58429389		II	SLC35F4 (-96798), C14orf37 (+189457)	
10	cg25966908	0.40	0.36	0.05	1.86E-03	Chr7:55516751		II	LANCL2 (+83611), VOPP1 (+123448)	
11	cg24565620	0.66	0.71	-0.05	4.30E-03	Chr2:194026		II	FAM110C (-147439), ACP1 (-70842)	
12	cg23795633	0.61	0.56	0.04	1.25E-03	Chr12:131417943		II	GPR133 (-20508), RAN (+61327)	
13	cg16454588	0.27	0.33	-0.06	4.80E-03	Chr5:140474184	PCDHB2	II	PCDHB2 (-52)	
14	cg19854521	0.41	0.47	-0.06	5.20E-03	Chr7:93520452	TFPI2	II	TFPI2 (-388)	
15	cg08269974	0.40	0.33	0.07	5.33E-03	Chr19:853054	ELANE	I	ELANE (+764)	
16	cg27408897	0.84	0.80	0.04	5.88E-04	Chr18:67136810	DOK6	II	DOK6 (+68527), CD226 (+487421)	
17	cg01824095	0.75	0.80	-0.05	3.97E-03	ChrX:40481859		II	ATP6AP2 (+41644), MED14 (+112944)	

Rank	Probe ID	Depressed Twin Mean	Co-twin Mean	βΔ	*P* Value	Hg19	Illumina Gene Annotation	Probe Type	Gene Annotation from GREAT (Distance from TSS)	Flagged Probes^
18	cg03880673	0.62	0.67	-0.05	3.01E-03	Chr16:65708104		II	CDH5 (-692420), CDH11 (-552186)	
19	cg20607798	0.30	0.24	0.06	5.76E-03	Chr8:58055168		I	FAM110B (-851944), IMPAD1 (-148739)	
20	cg15607708	0.31	0.27	0.05	4.87E-03	Chr19:54041308	ZNF331	I	ZNF331 (-231)	
21	cg24959134	0.45	0.41	0.04	4.92E-04	Chr12:97568165		II	NEDD1 (+267165)	
22	cg01756899	0.60	0.66	-0.06	6.11E-03	Chr16:1070137		II	SSTR5 (-52618), SOX8 (+38330)	
23	cg13365436	0.49	0.43	0.06	6.35E-03	Chr11:968650	AP2A2	II	AP2A2 (+42842), MUC6 (+68055)	
24	cg03551497	0.69	0.74	-0.05	4.37E-03	Chr5:4866009		II	ADAMTS16 (-274433)	
25	cg06188367	0.46	0.41	0.05	4.91E-03	Chr10:45359611		I	CXCL12 (-479067), TMEM72 (-47152)	
26	cg21829116	0.65	0.60	0.04	3.19E-03	Chr7:105337724	ATXN7L1	II	CDHR3 (-265932), EFCAB10 (-115749)	
27	cg14813083	0.57	0.53	0.04	7.77E-04	Chr11:18477280	LDHAL6A	II	LDHAL6A (-93)	
28	cg14089267	0.40	0.35	0.05	6.23E-03	Chr13:23412409		I	SGCG (-342650)	
29	cg23855093	0.68	0.64	0.04	1.03E-03	Chr3:100328713	GPR128	II	GPR128 (+281)	
30	cg12045829	0.55	0.51	0.04	1.42E-03	Chr17:7452926	TNFSF12-TNFSF13	II	TNFSF12-TNFSF13 (+552)	
31	cg12582426	0.49	0.53	-0.04	4.17E-03	Chr3:184227857		II	EPHB3 (-51729), CHRD (+129997)	
32	cg13988338	0.34	0.30	0.04	3.64E-03	Chr13:112986154		II	SPACA7 (-44496), SOX1 (+264242)	
33	cg02012721	0.71	0.75	-0.04	5.13E-03	Chr5:142197168	ARHGAP26	II	ARHGAP26 (+46877), NR3C1 (+586876)	*
34	cg21393587	0.69	0.74	-0.04	3.90E-03	ChrX:152342886		II	PNMA6A (+3979), MAGEA1 (+143229)	~
35	cg09342610	0.44	0.40	0.04	1.22E-03	ChrX:135333415	MAP7D3	I	MAP7D3 (+322)	

Rank	Probe ID	Depressed Twin Mean	Co-twin Mean	βΔ	*P* Value	Hg19	Illumina Gene Annotation	Probe Type	Gene Annotation from GREAT (Distance from TSS)	Flagged Probes^
36	cg09597400	0.34	0.30	0.04	1.59E-03	ChrX:149530886		I	MAMLD1 (-664)	
37	cg27150598	0.81	0.77	0.04	3.35E-03	Chr12:124325933	DNAH10	I	ZNF664 (-131828), DNAH10 (+78892)	
38	cg07364657	0.63	0.67	-0.05	6.49E-03	Chr8:97376970		II	SDC2 (-128911), PTDSS1 (+102804)	
39	cg00804245	0.54	0.49	0.05	7.52E-03	Chr14:106094144		I	TMEM121 (+101192), IGHE (+235317)	
40	cg24310913	0.70	0.65	0.04	5.30E-03	Chr15:63875268	USP3	II	USP3 (+78459), HERC1 (+250878)	
41	cg11157185	0.57	0.62	-0.04	5.18E-03	Chr1:59966003	FGGY	II	HOOK1 (-314529), FGGY (+203379)	
42	cg22108469	0.21	0.26	-0.05	7.26E-03	Chr5:174158984		II	MSX2 (+7410), DRD1 (+712178)	
43	cg02674474	0.40	0.36	0.04	2.08E-03	Chr20:9818397	PAK7	II	PAK7 (+1289), LAMP5 (+323393)	
44	cg23702848	0.65	0.70	-0.04	4.92E-03	Chr14:104172109	XRCC3	II	XRCC3 (+9713), KLC1 (+76585)	
45	cg25023198	0.42	0.46	-0.04	4.83E-03	Chr17:78323292	RNF213	II	ENDOV (-65674), RNF213 (+88626)	
46	cg13221767	0.64	0.68	-0.04	5.14E-03	Chr11:73721374	UCP3	II	UCP3 (-1093)	
47	cg10682833	0.62	0.57	0.05	7.98E-03	Chr19:49614538		I	LIN7B (-3079)	
48	cg19382572	0.61	0.66	-0.04	6.24E-03	Chr1:59765325	FGGY	II	HOOK1 (-515207), FGGY (+2701)	~
49	cg14163502	0.65	0.62	0.04	1.07E-03	Chr9:138619497	KCNT1	II	KCNT1 (+25467), CAMSAP1 (+179507)	
50	cg26786795	0.24	0.28	-0.04	1.85E-03	Chr14:20344394	OR4K2	II	OR4K2 (-32)	
51	cg04552811	0.65	0.70	-0.04	5.24E-03	Chr3:149708132		II	TSC22D2 (-418655), PFN2 (-19392)	
52	cg08450724	0.83	0.78	0.04	4.67E-03	Chr8:49172838		II	UBE2V2 (+251844), EFCAB1 (+475031)	
53	cg19390317	0.51	0.56	-0.05	6.90E-03	Chr21:43347995	C2CD2	II	PRDM15 (-48405), C2CD2 (+26003)	

Rank	Probe ID	Depressed Twin Mean	Co-twin Mean	βΔ	*P* Value	Hg19	Illumina Gene Annotation	Probe Type	Gene Annotation from GREAT (Distance from TSS)	Flagged Probes^
54	cg06219408	0.57	0.61	-0.04	3.80E-03	Chr8:143888878		II	GML (-27338), LY6D (-20871)	
55	cg07460734	0.80	0.76	0.04	2.21E-03	Chr19:57630153	USP29	II	USP29 (-1355)	
56	cg16533359	0.42	0.38	0.04	2.57E-03	Chr6:11710597		II	C6orf105 (+68682), TMEM170B (+172087)	
57	cg16714654	0.35	0.39	-0.05	7.25E-03	Chr14:69864029	ERH;SLC39A9	II	SLC39A9 (-1355), ERH (+991)	
58	cg10354232	0.80	0.76	0.04	1.39E-03	Chr2:133414633	LYPD1	II	LYPD1 (+13847), GPR39 (+240487)	
59	cg16757248	0.82	0.78	0.04	5.90E-03	Chr17:46872263	TTLL6	II	HOXB13 (-66153), TTLL6 (+22205)	
60	cg10027639	0.67	0.71	-0.04	4.28E-03	Chr11:23501632		II	SVIP (-650251)	
61	cg14241074	0.74	0.69	0.05	8.40E-03	Chr4:103752952	UBE2D3	II	UBE2D3 (-4245)	
62	cg11450541	0.78	0.74	0.04	4.93E-03	Chr13:24519920		I	SPATA13 (-214940), C1QTNF9B (-48776)	
63	cg07124642	0.36	0.40	-0.04	3.59E-03	Chr17:39991813	NT5C3L	II	KLHL10 (-2229), NT5C3L (+709)	
64	cg12419932	0.68	0.62	0.05	9.69E-03	Chr18:46069846	KIAA0427	II	CTIF (+4420), SMAD7 (+407234)	
65	cg14780070	0.28	0.32	-0.04	6.65E-03	Chr1:17951330	ARHGEF10L	II	ACTL8 (-130477), ARHGEF10L (+85001)	
66	cg06939402	0.66	0.62	0.04	2.75E-03	Chr13:114876521	RASA3	II	GAS6 (-309476), RASA3 (+21573)	**
67	cg26964112	0.67	0.62	0.05	9.87E-03	ChrX:69397273	DGAT2L6	II	DGAT2L6 (-62)	
68	cg13476044	0.67	0.63	0.04	8.36E-03	Chr19:50886715	POLD1	II	POLD1 (-877)	
69	cg22309696	0.32	0.27	0.04	7.60E-03	Chr2:172960993		II	DLX2 (+6484), DLX1 (+10786)	
70	cg26472922	0.49	0.54	-0.05	9.61E-03	Chr2:139803256		II	NXPH2 (-265446)	
71	cg27174822	0.59	0.54	0.05	1.01E-02	Chr16:2902830	PRSS22	II	PRSS30P (-10358), PRSS22 (+5340)	

Rank	Probe ID	Depressed Twin Mean	Co-twin Mean	βΔ	*P* Value	Hg19	Illumina Gene Annotation	Probe Type	Gene Annotation from GREAT (Distance from TSS)	Flagged Probes^
72	cg15169940	0.75	0.70	0.05	9.71E-03	Chr19:36604614	TBCB;POLR2I	I	TBCB (-1273)	
73	cg01249187	0.21	0.25	-0.04	6.95E-03	Chr16:1204617	CACNA1H	II	CACNA1H (+1377), TPSG1 (+70636)	
74	cg09210135	0.80	0.77	0.04	3.46E-04	Chr10:133858570		II	BNIP3 (-63136), JAKMIP3 (-59742)	
75	cg27272246	0.64	0.60	0.04	7.27E-04	Chr3:38664451	SCN5A	II	SCN5A (+10398), EXOG (+126689)	**
76	cg01507342	0.37	0.41	-0.05	9.10E-03	Chr17:65387096	PITPNC1	II	NOL11 (-326964), PITPNC1 (+13173)	
77	cg27245646	0.66	0.71	-0.05	9.74E-03	Chr3:127351538	PODXL2	II	ABTB1 (-40242), PODXL2 (+3500)	
78	cg08146156	0.78	0.82	-0.04	8.56E-04	Chr16:66918260	PDP2	II	PDP2 (+3878), CDH16 (+34626)	
79	cg12078929	0.18	0.14	0.04	7.24E-03	Chr22:42896688	SERHL	II	NFAM1 (-68288), RRP7A (+19140)	
80	cg21226735	0.59	0.63	-0.04	5.97E-03	Chr9:92807111		II	GADD45G (+587185), DIRAS2 (+597996)	
81	cg07458272	0.29	0.33	-0.04	4.78E-03	Chr19:34744396	KIAA0355	II	GPI (-111635), LSM14A (+81045)	
82	cg00053728	0.73	0.76	-0.04	5.19E-04	Chr2:3464167	TTC15	II	ADI1 (+59182), TRAPPC12 (+80722)	
84	cg01668653	0.76	0.72	0.04	6.54E-04	Chr6:35733710		I	C6orf126 (-10681), ARMC12 (+28852)	
83	cg05792650	0.65	0.69	-0.04	5.27E-03	Chr7:150901911		II	ASB10 (-16993), ABCF2 (+22405)	*
85	cg12161971	0.50	0.46	0.04	2.55E-03	Chr11:77907332	USP35	I	USP35 (+7475), GAB2 (+221535)	
86	cg13357714	0.64	0.69	-0.04	9.14E-03	Chr5:155807613	SGCD	II	PPP1R2P3 (-469935), SGCD (+53847)	
87	cg13058786	0.77	0.73	0.04	1.83E-03	Chr17:77097868	HRNBP3	I	ENGASE (+26850), RBFOX3 (+380811)	
88	cg01427142	0.74	0.70	0.04	4.43E-03	Chr7:144101769	NOBOX	II	NOBOX (+5550), ARHGEF5 (+49281)	
89	cg15062377	0.20	0.24	-0.04	2.35E-03	Chr1:200004205	NR5A2	II	FAM58BP (-178450), NR5A2 (+7436)	

Rank	Probe ID	Depressed Twin Mean	Co-twin Mean	βΔ	*P* Value	Hg19	Illumina Gene Annotation	Probe Type	Gene Annotation from GREAT (Distance from TSS)	Flagged Probes^
90	cg21252282	0.75	0.71	0.04	7.31E-03	Chr9:90439702		II	C9orf79 (-58069), CTSL3 (+51873)	
91	cg21146428	0.62	0.66	-0.04	5.60E-03	Chr14:101498343	MIR543	II	DIO3 (-529344), RTL1 (-147160)	
92	cg12740087	0.72	0.77	-0.04	9.19E-03	Chr1:224445738	NVL;MIR320B2	II	NVL (+72152), DEGS1 (+74829)	
93	cg02059755	0.71	0.75	-0.04	5.40E-03	ChrX:65383371	HEPH	II	HEPH (+939)	
94	cg04227631	0.63	0.67	-0.04	6.24E-03	ChrX:151562205	GABRA3;MIR767;MIR105-1	II	MAGEA5 (-275795), GABRA3 (+57625)	
95	cg13462219	0.49	0.53	-0.04	4.10E-04	Chr8:144649510	C8orf73	I	C8orf73 (+5417), GSDMD (+9034)	
96	cg00009292	0.24	0.28	-0.04	9.66E-03	Chr1:50882082		I	DMRTA2 (+7036), ELAVL4 (+307489)	
97	cg26880708	0.41	0.45	-0.04	7.26E-03	Chr11:114988776		II	CADM1 (+386464), FAM55B (+439577)	
98	cg02773588	0.74	0.71	0.04	1.95E-03	Chr3:48698519	CELSR3	I	TMEM89 (-39331), CELSR3 (+11461)	
99	cg27273125	0.60	0.65	-0.05	1.11E-02	ChrX:111919438	LHFPL1	II	LHFPL1 (+3936), ZCCHC16 (+593186)	
100	cg02776514	0.81	0.85	-0.04	2.23E-03	Chr11:121028926	TECTA	II	SC5DL (-134461), TECTA (+55552)	

^SNP located in probe extension site = *MAF < 0.05, **MAF > 0.05, unreactive probe = ~ as taken from Chen *et al.* 2013 (1).
DMP, differentially methylated probes; MAF, minor allele frequency; SNP, single nucleotide polymorphism; TSS, transcription start site.

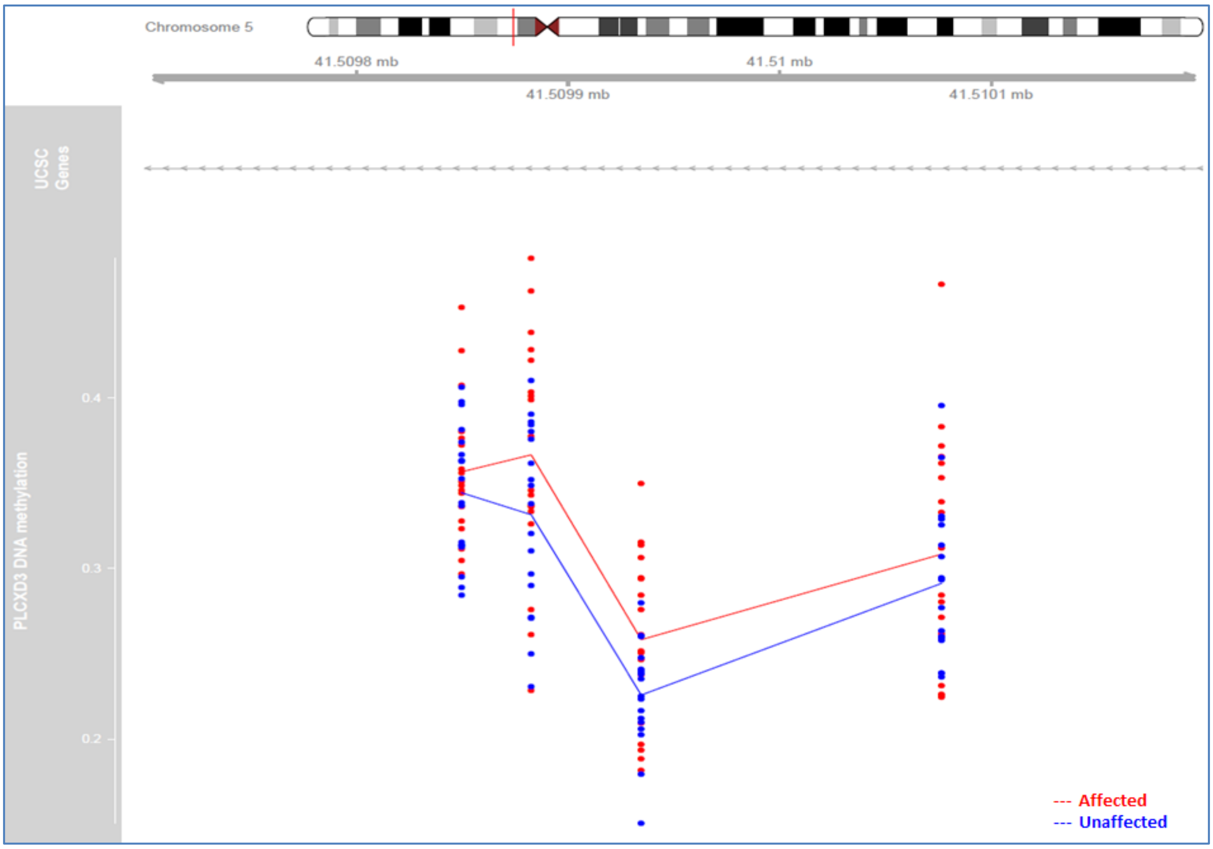

Figure S2. Illustrating the 300bp differentially methylated regions in the intron of gene *PLCXD3* located using the Illumina Methylation Analyzer regional analysis ($\Delta\beta$ = -0.024; p = 0.0005).

Table S4. Network Ingenuity Pathway Analysis: The top scoring functional networks of genes showing differential depression between the discordant twin pairs

	Associated Network Functions	Score
1	Cellular Assembly and Organization, Cellular Function and Maintenance, Cellular Movement	36
2	Cellular Movement, Cellular Development, Nervous System Development and Function	30
3	Carbohydrate Metabolism, Small Molecule Biochemistry, RNA Post-Transcriptional Modification	27
4	Endocrine System Development and Function, Lipid Metabolism, Molecular Transport	27
5	Hereditary Disorder, Neurological Disease, Psychological Disorders	25

Table S5. Functional Ingenuity Pathway Analysis: Top scoring diseases and disorders associated with genes showing differential depression between the discordant twin pairs

Name	*P* Value	# Molecules
Cardiovascular Disease	1.59E-04 - 2.34E-02	11
Organismal Injury and Abnormalities	1.59E-04 - 2.34E-02	15
Hypersensitivity Response	1.84E-04 - 1.57E-02	3
Inflammatory Response	1.84E-04 - 2.34E-02	9
Inflammatory Disease	9.04E-04 - 2.34E-02	13

Table S6. Functional Ingenuity Pathway Analysis: Top scoring physiological system development and function processes associated with genes showing differential depression between the discordant twin pairs

Name	*P* Value	# Molecules
Nervous System Development and Function	6.15E-05 - 2.34E-02	33
Tissue Morphology	6.15E-05 - 2.34E-02	22
Cardiovascular System Development and Function	3.65E-04 - 2.34E-02	12
Tissue Development	3.65E-04 - 2.34E-02	37
Embryonic Development	5.96E-04 - 2.34E-02	20

Table S7. IMA regional analysis results overview showing the top gene/region for each genomic classification

IMA Region	Gene/Region	*P* Value	Beta-Difference	Depressed Mean	Co-Twin Mean	Gene
TSS1500Ind	*F5*	0.00742	0.030	0.595	0.565	*F5*
TSS200Ind	*NPPA*	0.00226	0.030	0.756	0.726	*NPPA*
UTR5Ind	*FGF6*	0.00744	0.026	0.562	0.536	*FGF6*
EXON1Ind	*LDHC*	0.00271	0.031	0.311	0.280	*LDHC*
GENEBODYInd	*PNMA5*	0.00070	0.026	0.642	0.617	*PNMA5*
UTR3Ind	*POLR2I*	0.00971	0.049	0.751	0.702	*POLR2I*
ISLANDInd	chr5:41509783-41510166	0.00520	0.024	0.322	0.298	*PLCXD3*
NSHELFInd	chr10:134040198-134040418	0.00078	0.035	0.507	0.472	*STK32C*
NSHOREInd	chr18:67136907-67137248	0.00059	0.043	0.838	0.795	*DOK6*
SSHELFInd	chr4:103747631-103749392	0.00840	0.047	0.739	0.692	*UBE2D3*
SSHOREInd	chr1:101004471-101005885	0.01017	0.045	0.742	0.697	*GPR88*

IMA, Illumina Methylation Analyzer.

Supplementary Reference

1. Chen YA, Lemire M, Choufani S, Butcher DT, Grafodatskaya D, Zanke BW, *et al.* (2013): Discovery of cross-reactive probes and polymorphic CpGs in the Illumina Infinium HumanMethylation450 microarray. *Epigenetics* 8:203-209.
